# Mode of Patient Sexual Orientation and Gender Identity Disclosure and Receipt of Tailored Cancer Resources

**DOI:** 10.1001/jamanetworkopen.2025.38809

**Published:** 2025-10-23

**Authors:** Victor Basil, Charles Kamen, Austin R. Waters, N. F. N. Scout, Megan A. Mullins

**Affiliations:** 1 Department of Health Economics, Systems, and Policy, Peter O’Donnell Jr School of Public Health, University of Texas Southwestern Medical Center, Dallas; 2 Department of Surgery, University of Rochester Medical Center, Rochester, New York; 3 Department of Health Policy and Management, Gillings School of Global Public Health, University of North Carolina at Chapel Hill; 4 Lineberger Comprehensive Cancer Center, University of North Carolina at Chapel Hill; 5 National LGBT Cancer Network, Providence, Rhode Island; 6 Department of Internal Medicine, University of Texas Southwestern Medical Center, Dallas; 7 Harold C. Simmons Comprehensive Cancer Center, University of Texas Southwestern Medical Center, Dallas

## Abstract

**Importance:**

For lesbian, gay, bisexual, transgender, queer, questioning, and other (LGBTQ+) individuals with cancer, disclosing sexual orientation and gender identity (SOGI) in a safe clinical environment can improve survivorship outcomes and satisfaction. Although SOGI disclosure can facilitate patient-centered care, little is known about whether SOGI information is being used to improve care or how use may vary by mode of SOGI disclosure.

**Objective:**

To assess whether the mode of SOGI disclosure (self-disclosed, clinic collected, not disclosed, or other) is associated with receipt of clinical resources tailored to LGBTQ+ individuals among cancer survivors.

**Design, Setting, and Participants:**

This cross-sectional study uses data from the OUT National Cancer Survey, a web-based survey of LGBTQ+ adults with a history of cancer in the US, collected from September 2020 through March 2021. The study comprised a convenience sample of 2342 LGBTQ+ cancer survivors.

**Main Outcome and Measures:**

Multivariable logistic regression was used to assess the association between SOGI disclosure mode and receipt of at least 1 clinical resource tailored to LGBTQ+ individuals related to mental health, tobacco use, alcohol use, physical activity, or survivorship care. The primary exposure was mode of SOGI disclosure. Covariates included age, cancer type, current cancer status, and region.

**Results:**

This national sample of 2342 LGBTQ+ cancer survivors (mean [SD] age, 58.4 [16.7] years; 1428 assigned male at birth [61%]) included 79 Black participants (3%), 136 Hispanic participants (6%), 2019 White participants (86%), 64 multiracial participants (3%), and 90 participants of other race or ethnicity (4%). A total of 1394 participants (60%) identified as cisgender male, and 775 (33%) as cisgender female, and the most common sexual orientation was gay (1272 [54%]). Participants reported many cancer types, with genitourinary cancers being most common (523 [22%]). Most respondents (1798 [77%]) indicated that their care team knew their LGBTQ+ identity, with disclosure most often via self-disclosure (804 [34%]) or clinic collection (944 [42%]). Only 174 (7%) received an LGBTQ+ survivorship care plan, and 892 (38%) received at least 1 resource tailored to LGBTQ+ individuals. Compared with those with clinic-collected SOGI disclosure, those who did not disclose SOGI information (odds ratio [OR], 0.58 [95% CI, 0.44-0.78]) or who self-disclosed SOGI information (OR, 0.84 [95% CI, 0.69-1.02]) had lower odds of receiving any tailored resources.

**Conclusions and Relevance:**

In this cross-sectional study of SOGI disclosure and use, few LGBTQ+ cancer survivors received tailored clinical resources despite high rates of SOGI disclosure. Clinical use of SOGI information remained limited, especially when it was not collected systematically. Efforts should prioritize both safe SOGI data collection and its integration into patient-centered care.

## Key Points

**Question:**

Is the mode of sexual orientation and gender identity (SOGI) disclosure associated with receipt of clinical resources tailored to LGBTQ+ individuals among cancer survivors?

**Findings:**

In this cross-sectional study of a national sample of 2342 LGBTQ+ cancer survivors, those who self-disclosed or did not disclose SOGI information were less likely to receive at least 1 LGTBQ+ clinical resource compared with those who had SOGI information collected by the clinic.

**Meaning:**

This study suggests that continued efforts for systematic SOGI data collection and use are needed to improve delivery of tailored resources and care for LGBTQ+ people with cancer.

## Teaser

This cross-sectional study assesses whether the mode of sexual orientation and gender identity disclosure was associated with receipt of clinical resources tailored to LGBTQ+ individuals among cancer survivors in the US.

## Introduction

Patient-centered care, a core component of high-quality cancer care, is not only responsive to patient needs and values but is emotionally supportive, allows for involvement of loved ones, and supports informed patient decision-making with information and education.^1,2,3^ To deliver patient-centered care, clinical care teams need to know salient patient identities and relationships. For lesbian, gay, bisexual, transgender, queer, questioning, and other (LGBTQ+) patients, disclosing this information may not always feel safe or welcomed, but evidence shows that LGBTQ+ patients have more satisfaction with care and better survivorship outcomes when they can safety disclose their identities with their clinical care team.^4,5,6,7,8^

Although identity disclosure may happen informally in a clinical encounter, lack of systematic processes to elicit this information respectfully from all patients can result in unequal targeting of patients based on appearance or other staff perceptions, and it burdens patients with fear of disclosure and negative reactions.^4,9,10^ In addition, ad hoc identity disclosure may not be documented at all, or if it is documented, not in a place where it is easily identifiable and useful to clinical staff.^9^ Because discrete data fields facilitate systematic collection, a 2015 policy mandated that electronic health record systems in the US should have the capacity to collect sexual orientation and gender identity (SOGI) data by 2018 under the US Meaningful Use Stage 3 incentive program.^9,11,12^ Although rates of SOGI data collection have increased in recent years, most oncology practices are still not systematically collecting these data from patients, although SOGI data still may be disclosed and used in visits.^12,13^ Given the volume of information in the electronic health record and the lack of SOGI data awareness among clinical teams, it is unknown whether systematic SOGI data collection or informal SOGI disclosure are resulting in increased use of SOGI data in clinical practice.^9,14^ There are numerous ways SOGI data can be used to inform clinical practice, including informing the language used in clinical encounters, referrals to LGBTQ+-specific support groups or clinicians, or provision of clinical resources (such as education about substance use, mental health, or physical activity) that are tailored to the experience of LGBTQ+ patients.^15,16,17^

Several studies have shown that LGBTQ+ individuals with cancer have unmet informational and educational needs and a desire for LGBTQ+-tailored cancer resources.^18,19,20,21^ Tailored resources can address critical population-specific needs and improve clinical outcomes, satisfaction, and engagement.^15,16,20,22,23,24^ To understand the association between mode of SOGI disclosure and use of SOGI data to provide LGBTQ+-tailored resources to patients, we analyzed data from the OUT National Cancer Survey, a national survey of LGBTQ+ cancer survivors.^25^ We evaluated how survivors’ care teams knew their SOGI and whether the mode of identity disclosure was associated with receipt of LGBTQ+-tailored clinical resources for tobacco cessation, mental health, physical activity, alcohol consumption, or cancer survivorship care.

## Methods

### Study Setting and Participants

The OUT National Cancer Survey, conducted from September 2020 through March 2021, has previously been described in detail.^25,26,27^ In brief, adults (≥18 years) with a self-reported history of a cancer diagnosis who identify as LGBTQ+ and live in the US were eligible and recruited for survey response via online platforms and targeted ad campaigns.^25^ From an initial sample of 4517 respondents, we excluded ineligible individuals, including those who did not report having cancer (n = 488), who did not identify as LGBTQ+ (n = 49), who were not at least 18 years of age (n = 9), and who were outside the US (n = 28) (eFigure in Supplement 1). Because this was a web-based survey, we also took additional steps to identify and exclude fraudulent responses from survey bots and other illegitimate respondents.^28^ To identify fraudulent responses, we deployed a Stata, version 17 (StataCorp LLC) module (checkipaddresses) that queries an IP address verification service to determine if participants were located outside of the US or used a virtual private network or server to mask their true location.^29,30^ A total of 2175 responses were excluded due to ineligibility (n = 574) or being flagged as fraudulent (n = 1601), resulting in a final analytic sample of 2342 LGBTQ+ cancer survivors. The protocol and survey were approved by the Western institutional review board. After confirming eligibility and reading the informed consent form, participants electronically consented to participate. Participation was voluntary and anonymous, and participants were not provided with incentives for participation. This report followed the Strengthening the Reporting of Observational Studies in Epidemiology (STROBE) reporting guideline for cross-sectional studies.^31^

### Outcome

The primary outcome, receipt of LGBTQ+-tailored clinical resources, was assessed by the following survey questions: “Does your post-treatment care plan include resources for LGBTQ+ individuals?” “Have you ever received resources to help you stop using tobacco that were developed for LGBTQ+ individuals?” “Have you ever received resources related to alcohol consumption developed for LGBTQ+ individuals?” “Have you ever received resources related to physical activity developed for LGBTQ+ individuals?” “Have you ever received resources related to mental health developed for LGBTQ+ individuals?” Response options were “yes,” “no,” and “don’t know/prefer not to answer.” For smoking and alcohol resource reporting, we assessed receipt among those with a smoking history or drinking history. From item-specific responses, a composite variable for receipt of tailored resources (yes or no) was created to indicate receipt of at least 1 type of tailored resource.

### Exposure

The primary exposure of interest, mode of SOGI disclosure, was assessed by the survey question “Were/are any of your cancer healthcare professionals aware of your LGBTQ+ identity?” Response options were “yes,” “no,” and “don’t know/prefer not to answer.” Respondents answering “no” were categorized as “did not disclose.” Respondents answering “yes” were asked, “How did this generally come about? (select all that apply).” Responses were grouped into categories: clinic collected (includes responses: “Patient or medical forms gave the option for me to say,” “Health professional asked during a consultation,” “Nurses asked,” and “It is embedded in my medical information”), self-disclosure (includes responses: “Self-disclosed during a consultation”), and don’t know, missing, or other (includes responses: “People can usually tell by my appearance,” “Someone else told the healthcare professional,” and “Something else”). If respondents selected “other” and provided their own text, we assigned the response to the most pertinent category.

### Covariates

Other variables examined include respondent clinical and sociodemographic characteristics: sex assigned at birth (male, female, or prefer not to share), physical differences in sexual anatomy at birth (intersex; yes, no, or prefer not to share), gender (female, male, nonconforming or nonbinary [includes genderqueer], transgender, other, or prefer not to share), sexual orientation (lesbian, gay, bisexual, another orientation [includes asexual spectrum, pansexual, queer, and another orientation], or multiple orientations), age when diagnosed with cancer and age at time of survey completion (18-44 years, 45-64 years, ≥65 years, or missing), region of US where patient resides (Northeast, Midwest, South, West, or unknown), cancer type (blood, breast, gastrointestinal, genitourinary, gynecologic, lung, other, missing, or unknown), currently have health insurance (yes, no, or unknown), highest level of education completed (some high school, high school diploma, some college or vocational school, college or vocational school degree or certificate, graduate school, unknown or missing), ethnicity (Hispanic, non-Hispanic, or prefer not to share), current cancer diagnosis (yes, no, or unknown), and currently participating in treatment (yes, no, unknown, or no current diagnosis). Race was collapsed into Black, White, another racial minority (including Alaska Native, American Indian, Asian or Asian American, Middle Eastern or North African, Native Hawaiian or Pacific Islander, and a text value for “a racial identity not listed here”), multiracial, and prefer not to share. Participants self-identified their race in the survey. Race and ethnicity data were collected to further understand and describe the participants in this study. LGBTQ+ identities, their salience, and their disclosure can differ across racial and ethnic identities.

### Statistical Analysis

Bivariate analyses were conducted using χ^2^ tests to assess unadjusted associations between SOGI disclosure mode and receipt of each individual tailored resource (tobacco cessation, alcohol use, physical activity, mental health, posttreatment care, and general LGBTQ+ cancer information). A multivariable logistic regression model was used to examine the association between mode of SOGI disclosure and receipt of at least 1 LGBTQ+-tailored clinical resource, adjusted for US region of residence, age at cancer diagnosis, current cancer diagnosis status (yes or no), and type of cancer. Missing values for variables that did not require an answer to submit the assessment were listed as missing or were collapsed into the category “unknown” with responses “other” and “don’t know.” A 2-sided *P* < .05 was statistically significant. Analyses were conducted using SAS, version 9.4 (SAS Institute Inc).

## Results

This national sample of 2342 LGBTQ+ cancer survivors (mean [SD] age, 58.4 [16.7] years; 1428 assigned male at birth [61%] and 900 assigned female at birth [38%]) included 79 Black participants (3%), 136 Hispanic participants (6%), 2019 White participants (86%), 64 multiracial participants (3%), and 90 participants of other race or ethnicity (4%) (Table 1). More than half of the respondents were cisgender males (1394 [60%]), followed by cisgender females (775 [33%]), nonbinary or nonconforming individuals (101 [4%]), transgender individuals (43 [2%]), and individuals of another gender (29 [1%]). Most participants reported gay sexual orientation (1272 [54%]), followed by lesbian (581 [25%]), multiple orientations (246 [11%]), another orientation (127 [5%]), and bisexual (116 [5%]). Most participants received a diagnosis of cancer between the ages of 45 and 64 years (1438 [61%]). Almost one-fourth of respondents (514 [22%]) had cancer at the time of survey completion. Respondents reported many different types of cancer, with genitourinary cancers being the most common (523 [22%]).

**Table 1.** Demographic and Clinical Characteristics of LGBTQ+ Cancer Survivors Participating in the OUT National Cancer Survey

Characteristic	No. (%) (N = 2342)
Sex assigned at birth	
Female	900 (38)
Male	1428 (61)
I prefer not to share this information	14 (1)
Physical differences in sexual anatomy at birth (intersex)	
No	2277 (97)
Yes	49 (2)
I prefer not to share this information	16 (1)
Gender	
Female	775 (33)
Male	1394 (60)
Nonconforming or nonbinary	101 (4)
Transgender	43 (2)
Another gender	29 (1)
Sexual orientation	
Bisexual	116 (5)
Gay	1272 (54)
Lesbian	581 (25)
Multiple orientations	246 (11)
Another orientation	127 (5)
Age at cancer diagnosis, y	
≤18	39 (2)
19-44	572 (24)
45-64	1438 (61)
≥65	256 (11)
Missing	37 (2)
Age at time of survey, y	
18-44	270 (12)
45-64	1281 (55)
≥65	665 (28)
Missing	126 (5)
Region of US where patient resides	
Midwest	382 (16)
Northeast	400 (17)
South	574 (25)
West	536 (23)
Unknown	450 (19)
Types of cancer	
Blood	305 (13)
Breast	388 (17)
Gastrointestinal	310 (13)
Genitourinary	523 (22)
Gynecologic	192 (8)
Lung	102 (4)
Other	504 (22)
Missing	18 (1)
Currently have health insurance	
No	70 (3)
Yes	2164 (92)
Unknown	108 (5)
Highest level of education completed	
Graduate school	885 (38)
College or vocational school degree or certificate	864 (37)
Some college or vocational school	381 (16)
High school diploma	75 (3)
Some high school	15 (1)
I prefer not to share this information	7 (<1)
Unknown	115 (5)
Race	
Black	79 (3)
White	2019 (86)
Other^a^	90 (4)
Multiracial	64 (3)
I prefer not to share this information	33 (2)
Missing	57 (2)
Ethnicity	
Hispanic	136 (6)
Non-Hispanic	2078 (89)
Missing	128 (5)
Currently have cancer	
No	1691 (72)
Yes	514 (22)
Unknown	137 (6)
Currently receiving cancer treatment	
No	153 (7)
Yes	354 (15)
Unknown	144 (6)
No current diagnosis	1691 (72)
Mode of LGBTQ+ identity disclosure	
Clinic collected	994 (42)
Self-disclosed	804 (34)
Did not disclose	288 (12)
Don’t know, missing, or other	256 (11)

Abbreviation: LGBTQ+, lesbian, gay, bisexual, transgender, queer, questioning, and other.

^a^ Other included Alaska Native, American Indian, Asian or Asian American, Middle Eastern or North African, Native Hawaiian or Pacific Islander, and a text value for “a racial identity not listed here.”

Most respondents (1798 [77%]) indicated that their care team knew their LGBTQ+ identity, with disclosure most often via self-disclosure (804 [34%]) or clinic collection (944 [42%]). Table 2 presents data on LGBTQ+-tailored resource receipt by the mode of SOGI disclosure. The most commonly reported tailored resource was the LGBTQ+ mental health resource (667 [28%]) and the LGBTQ+-tailored resource for tobacco cessation (95 of 342 reported smokers [28%]). Only 174 respondents (7%) received an LGBTQ+ survivorship care plan, and 892 (38%) received at least 1 resource tailored to LGBTQ+ individuals. Overall, more patients whose SOGI data were collected by the clinic received at least 1 tailored resource (414 [18%]), compared with those who self-disclosed (298 [13%]) or did not disclose (88 [4%]) (*P* < .001). Participants who did not disclose their identity received the fewest tailored resources across all resource types.

**Table 2.** Receipt of LGBTQ+ Tailored Resources by Disclosure Mode in the OUT National Cancer Survey

Disclosure mode	Respondents, No. (%)											
	Survivorship plan (n = 1056)^a^		Tobacco resource (n = 342)^b^		Alcohol resource (n = 767)^c^		Physical activity resource (n = 2342)^d^		Mental health resource (n = 2342)^d^		Any resource (n = 2342)^d^	
	No	Yes	No	Yes	No	Yes	No	Yes	No	Yes	No	Yes
Clinic collected	397 (38)	102 (82)	100 (29)	36 (11)	297 (39)	50 (7)	857 (38)	103 (5)	627 (28)	318 (14)	580 (25)	414 (18)
Self-disclosed	305 (29)	57 (5)	95 (28)	40 (12)	239 (31)	29 (4)	696 (31)	63 (3)	203 (9)	70 (3)	506 (22)	298 (13)
Did not disclose	98 (10)	9 (1)	33 (10)	9 (3)	64 (9)	11 (1)	262 (31)	16 (1)	168 (8)	59 (3)	200 (9)	88 (4)
Do not know, missing, or other	82 (8)	6 (1)	19 (6)	10 (3)	66 (9)	11 (1)	217 (9)	19 (1)	537 (24)	220 (10)	164 (7)	92 (4)
*P* value^e^	.001		.61		.57		.05		.02		.004	

Abbreviation: LGBTQ+, lesbian, gay, bisexual, transgender, queer, questioning, and other.

^a^ Among 1056 respondents who received a care plan.

^b^ Among 342 respondents who reported smoking.

^c^ Among 767 respondents who reported drinking alcohol.

^d^ Among 2342 total survey respondents.

^e^ χ^2^ Statistics were used to generate *P* value.

In the adjusted model, participants who did not disclose their SOGI information had significantly lower odds of receiving at least 1 tailored resource compared with those whose clinic intentionally collected SOGI information (odds ratio, 0.58 [95% CI, 0.44-0.78]) (Figure). Although not a statistically significant difference, those who self-disclosed also had lower odds of tailored resource receipt compared with those whose clinic intentionally collected SOGI information (odds ratio, 0.84 [95% CI, 0.69-1.02]).

**Figure.**
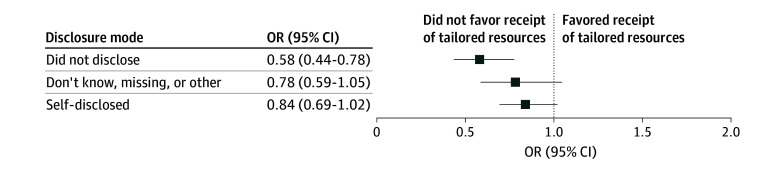
Adjusted Odds Ratios (ORs) for Receipt of a Lesbian, Gay, Bisexual, Transgender, Queer, Questioning, and Other–Tailored Resource for OUT National Cancer Survey Respondents Who Disclosed Sexual Orientation and Gender Identity to Their Clinical Team vs Those Who Did Not The logistic regression model includes adjustments for US region, cancer type, age at cancer diagnosis, and current cancer, allowing for estimation of the association between identity disclosure category and receipt of tailored resources while controlling for these covariates.

## Discussion

In this national study of LGBTQ+ cancer survivors, most respondents (approximately 90%) reported that their care team was aware of their LGBTQ+ identity, but only one-third (38%) received an LGBTQ+-tailored resource. Receipt of tailored resources was most common among individuals whose clinic collected their SOGI information. With much of the literature focused on SOGI data collection, to our knowledge, this is the first study to assess the relevance of mode of SOGI disclosure or its use in provision of tailored clinical resources for LGBTQ+ patients with cancer, a critical first step toward precision cancer care for LGBTQ+ patients.

As demonstrated in other studies, continued efforts to implement clinic-led SOGI data collection are needed, as less than half (42%) of the reported SOGI disclosures were clinic collected.^12,13,32,33^ Approximately one-third of identity disclosures were self-disclosed without clinic solicitation. Although identity disclosure may happen easily in the course of a clinical encounter, it can also cause additional distress and emotional burden to patients and their caregivers if they deliberate whether or not to disclose their identity or fear negative reactions.^4,10^ The large proportion of patients disclosing their identities illustrates the salience of LGBTQ+ cultural competence and general familiarity with SOGI among clinical staff to ensure these disclosures are handled safely and respectfully.^9,34,35,36,37^

Our findings suggest efforts to use SOGI more intentionally in clinical care are needed. Disclosure of SOGI has been associated with improved rates of engagement in preventive care, treatment adherence, and satisfaction with care.^15,23,32^ Although safe disclosure may facilitate better outcomes through a patient feeling seen and free to bring themselves holistically to their clinical care, it can also be used to deliver tailored resources that patients value. In a prior analysis of OUT Cancer Survey data, Burnett and colleagues^19^ found that 57% of respondents felt that a survivorship care plan with LGBTQ+ information was important, and 80% of respondents agreed that LGBTQ+ mental health resources were valuable; however, in our study, approximately 10% of survivorship care plan recipients received tailored information, and approximately one-third received a tailored mental health resource. These rates were higher when clinics intentionally collected SOGI information compared with when patients self-disclosed or did not disclose SOGI information. Intentional data collection populated into discrete fields may be more visible within the electronic health record display than identities buried in clinical notes.^38^ This information could also help connect patients to tailored resources, not only written materials with LGBTQ+ content but also caregiver resources for LGBTQ+ caregivers, support groups, inclusive clinician lists for ancillary services such as mental health and counseling, or inclusive and welcoming gyms for physical activity to ameliorate cancer fatigue and improve mental health.^10,39,40^ Similar to the requisite trainings on how to collect SOGI data, training and other supports are needed to support clinicians in learning how to use SOGI data.^32^

### Limitations

The study has some limitations. Our online national survey approach afforded a substantial sample size from diverse US locations and enabled participation from people who may not be actively engaged with a health care system. However, online surveys without identity verification are susceptible to fraudulent responses. It is possible that our method of removing potential fraudulent responses could have removed legitimate responses from individuals who were using a virtual proxy network, and we may have failed to capture all true fraudulent responses. Given the anonymity of the survey and our available data, we were unable to conduct a bias analysis. Future studies should warn potential participants to turn off their virtual proxy network prior to participating to avoid this issue. Second, because we used convenience sampling without a known denominator, we cannot ascertain a response rate or selection bias among those who participated. Despite intentional recruitment to produce a diverse sample, the sample included many White participants who were assigned male at birth and highly educated, which may impact generalizability. We also could not link to any medical records to verify self-reports of cancer. Systematic SOGI data collection across clinical settings is critical to facilitate future large-scale population-based surveys of LGBTQ+ cancer survivors. Given the lack of generalizable information available on LGBTQ+ cancer survivors, this large sample offers an important starting point for understanding how SOGI data are being used to provide tailored resources.

## Conclusions

In this cross-sectional study of a national sample of LGBTQ+ cancer survivors, identity disclosure was common, but using that disclosure to provide tailored resources was not, especially in settings in which disclosure was not through clinic collection of SOGI data. Future studies should focus not only on SOGI data collection but also clinical use of SOGI data and the effectiveness of SOGI-tailored resources for improving patient outcomes. Systematic SOGI data collection will facilitate survey studies in the future for this understudied but growing population to better understand health disparities and outcomes for sexual and gender minority patients, contributing to the development of high-quality, patient-centered care.

## Notes

Supplement 1. **eFigure.** Participant Flow

Supplement 2.
Data Sharing Statement
