# Supplementary material for: Mode of Patient Sexual Orientation and Gender Identity Disclosure and Receipt of Tailored Cancer Resources
Source: JAMA Netw Open. 2025 Oct 23;8(10):e2538809. doi: 10.1001/jamanetworkopen.2025.38809 (PMC12550635; doi:10.1001/jamanetworkopen.2025.38809)
Supplement: Supplement 1. — eFigure. Participant Flow [file jamanetwopen-e2538809-s001.pdf]

Supplementary Online Content

Basil V, Kamen C, Waters AR, Scout NFN, Mullins MA. Mode of patient sexual orientation and gender identity disclosure and receipt of tailored cancer resources. *JAMA Netw Open.* 2025;8(10):e2538809. doi:10.1001/jamanetworkopen.2025.38809

eFigure. Participant Flow

This supplementary material has been provided by the authors to give readers additional information about their work.

© 2025 Basil V et al. *JAMA Network Open.*

eFigure. Participant Flow

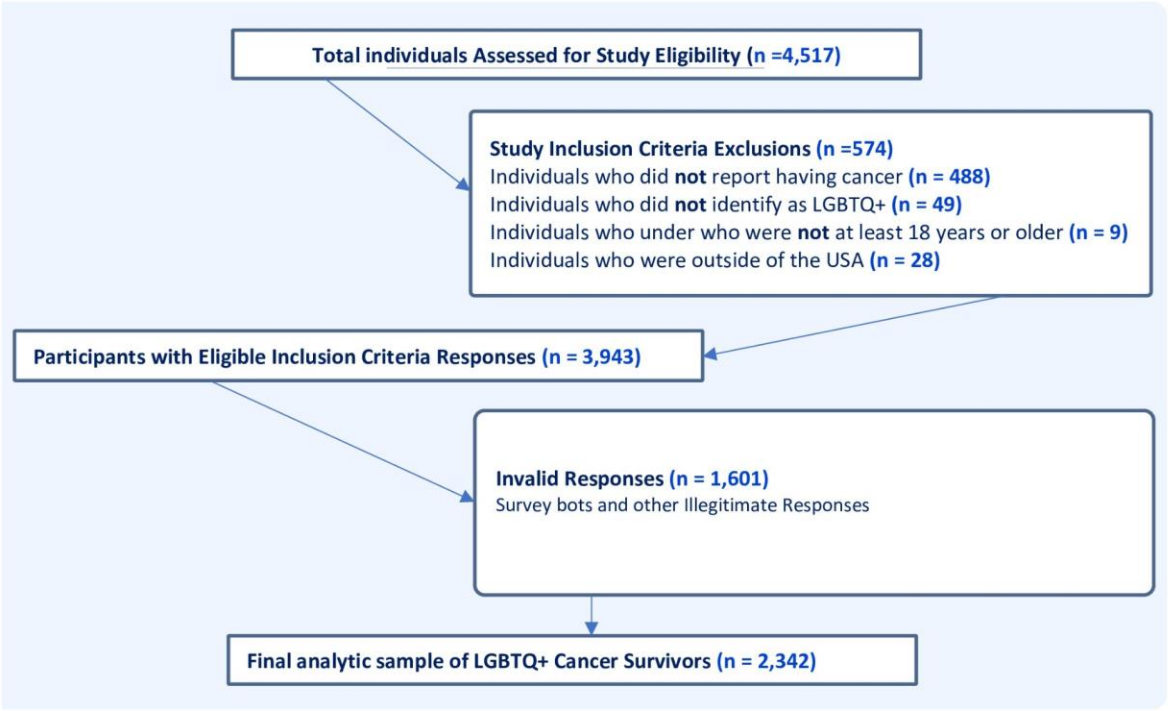

© 2025 Basil V et al. *JAMA Network Open.*
