# Supplementary material for: Mode of Patient Sexual Orientation and Gender Identity Disclosure and Receipt of Tailored Cancer Resources
Source: JAMA Netw Open. 2025 Oct 23;8(10):e2538809. doi: 10.1001/jamanetworkopen.2025.38809 (PMC12550635; doi:10.1001/jamanetworkopen.2025.38809)
Supplement: Supplement 2. — Data Sharing Statement [file jamanetwopen-e2538809-s002.pdf]

Data Sharing Statement

Basil. Mode of Patient Sexual Orientation and Gender Identity Disclosure and Receipt of Tailored Cancer Resources. *JAMA Netw Open*. Published October 23, 2025. doi:10.1001/jamanetworkopen.2025.38809

Data
Data available: No
